# Can Gas Replace Protein Function? CO Abrogates the Oxidative Toxicity of Myoglobin

**DOI:** 10.1371/journal.pone.0104075

**Published:** 2014-08-11

**Authors:** Elena A. Sher, Alan Y. Sholto, Mati Shaklai, Nurith Shaklai

**Affiliations:** 1 Department of Human Genetics and Biochemistry, Sackler Faculty of Medicine, Tel Aviv University, Tel Aviv, Israel; 2 Department of Hematology, Sackler Faculty of Medicine, Tel Aviv University, Tel Aviv, Israel; Universidade Federal do Rio de Janeiro, Brazil

## Abstract

Outside their cellular environments, hemoglobin (Hb) and myoglobin (Mb) are known to wreak oxidative damage. Using haptoglobin (Hp) and hemopexin (Hx) the body defends itself against cell-free Hb, yet mechanisms of protection against oxidative harm from Mb are unclear. Mb may be implicated in oxidative damage both within the myocyte and in circulation following rhabdomyolysis. Data from the literature correlate rhabdomyolysis with the induction of Heme Oxygenase-1 (HO-1), suggesting that either the enzyme or its reaction products are involved in oxidative protection. We hypothesized that carbon monoxide (CO), a product, might attenuate Mb damage, especially since CO is a specific ligand for heme iron. Low density lipoprotein (LDL) was chosen as a substrate in circulation and myosin (My) as a myocyte component. Using oxidation targets, LDL and My, the study compared the antioxidant potential of CO in Mb-mediated oxidation with the antioxidant potential of Hp in Hb-mediated oxidation. The main cause of LDL oxidation by Hb was found to be hemin which readily transfers from Hb to LDL. Hp prevented heme transfer by sequestering hemin within the Hp-Hb complex. Hemin barely transferred from Mb to LDL, and oxidation appeared to stem from heme iron redox in the intact Mb. My underwent oxidative crosslinking by Mb both in air and under N_2_. These reactions were fully arrested by CO. The data are interpreted to suit several circumstances, some physiological, such as high muscle activity, and some pathological, such as rhabdomyolysis, ischemia/reperfusion and skeletal muscle disuse atrophy. It appear that CO from HO-1 attenuates damage by temporarily binding to deoxy-Mb, until free oxygen exchanges with CO to restore the equilibrium.

## Introduction

Hemoglobin (Hb) and myoglobin (Mb) are the two major respiratory hemoproteins (RH) burdened with the task of maintaining aerobic life: Hb shuttles oxygen from the lungs to tissues, while Mb maintains a store of oxygen in muscles, ready for use. Structural similarities in their globin (gl) backbones and a common ferrous heme iron at their active site indicate similar functionalities [1], [2]. Both globins undergo conformational changes between oxy and deoxy states, and both possess a ferrous iron (Fe^II^) at the center of a heme group to provide the conditions for oxygen binding. The oxygen binding task of RH necessitates a heme ferrous iron, even though the ferric iron valency is the more stable and indeed is used as ground state of other metabolic hemoproteins. To keep the iron in its ferrous state, a reducing environment is maintained both in myocyte and in the nucleus-free red blood cell (RBC). Should a cell rupture, the expelled oxy-ferrous RH is no longer insulated within a protective reducing environment and spontaneously undergoes auto-oxidation to the ferric (Fe^III^) form [3]. The auto-oxidation reaction generally yields a reactive oxygen species, like hydrogen peroxide, which goes on to wreak oxidative havoc in the cell: H_2_O+gl-Fe^II^-O_2_→gl-Fe^III^+H_2_O_2_. This process is well documented in the literature [4]. Moreover, in pathologies with increased hemolysis like malaria [5] or sickle cell anemia auto-oxidation is increased [1], [3]. Both RH also possess redox activity based on Fe^III^ to Fe^IV^ redox [2]–[4].

Despite structural and functional similarities, major differences exist in the cellular habitats and turnover of the two proteins. It has been pointed out that deleterious Hb redox activity is dominated by its tendency to release heme once oxidized, while Mb redox activity tends to be based more on the intact hemoprotein [5], [6]. Besides structural differences, their physiology and modus operandi differ: Hb is sequestered into a RBC lacking a nucleus and containing essential reducing enzymes. RBCs are prone to frictional damage as they journey through narrow capillaries, and, since the cell wall may get damaged, Hb may be released. Cell-free Hb is oxidatively destructive [1]–[7], necessitating the average RBC to remain in regular circulation for only ∼12 weeks (and much shorter in pathological cases), after which it is replaced by a newly-formed one. In contrast, myocytes have a low turnover rate, and only if rhabdomyolysis occurs, extracellular Mb accumulates in the kidneys, causing renal failure [8], [9].

Two plasma proteins Haptoglobin (Hp) and Hemopexin (Hx) scavenge extracellular Hb and heme respectively. Hp has an extremely high affinity for Hb and, once bound, escorts it to the liver where the Hp-Hb complex binds to the scavenger receptor, CD163 [10]–[12]. Hx has a high and specific affinity for hemin (heme-Fe^III^) and, after binding, clears it out via the CD91 receptor in the liver. The two plasma proteins work in tandem [1]. At the liver, the enzyme Heme Oxygenase (HO) converts hemin to biliverdin, iron and carbon monoxide (CO) [13]–[15]. Intense research from recent decades has shown that genes for the induced enzyme, HO-1, are expressed in most cells, and that enzyme levels are elevated under conditions which weaken the heme-globin bond, like stress, oxidative stress, sepsis, malaria, hypertension. In cases with increased hemolysis, like sickle cell anemia which was intensively studied, it was established that CO produced from HO-1 induced by the loosely bound (free) hemin, is in fact salutary [16]–[18]. Another hemolytic situation studied this concern is malaria, where added or endogenously formed CO has been demonstrated therapeutic [19], [20]. While much research has concentrated on the body's defense mechanisms against Hb and its breakaway heme, practically nothing is known about the ways in which the body deals with oxidatively toxic Mb. Mb escapes into the acellular space as a consequence of muscle trauma and can also wreak harm in the cell. Oxidative damage, once thought to be the result of free iron released from Mb, was later studied by observing the Mb-induced LDL oxidation model and found to be the consequence of intact Mb redox cycling activity [21]. A study, based on a rat-model of rhabdomyolysis, discovered that Mb-mediated oxidative damage in the kidney is followed by the rapid induction of HO-1 [22]. The same group reported that, by induction of HO-1 prior to rhabdomyolysis via administration of either Hb or hemin, full protection was provided against the damage [23]. These data imply that the enzyme HO-1 and/or its products, bilirubin and CO, provide protection. Moreover, one of our previous studies indicated that a mutual presence of CO and peroxide, hydrophilic as H_2_O_2_ or hydrophobic as in oxidized LDL, blocks oxidative damage by Hb and Mb [24].

Based on the information available, we reasoned that CO might act as a specific inhibitor, arresting damage caused by Mb. Hence, the current study was devoted to a comparison of Hp shielding Hb and the protection afforded by CO against Hb and Mb. Importantly, care was taken to maintain conditions as close to *in vivo* as possible. These include: mild oxidative conditions comprising a few micromolar H_2_O_2_, a low concentration (a few µM) of hemoprotein and no free oxygen [25].

## Materials and Methods

All experimental protocols were approved by the Institutional Animal Care and Use Committee (IACUC) at the Hebrew University of Jerusalem, which adheres to the Israeli guidelines which follows the NIH/USA animal care and use protocols.

Blood donors for the preparation of LDL and Hb (see below), were fully informed concerning this study and signed a written consent form; ethical approval for this study was obtained through the research ethics board of Tel-Aviv University.

Frozen muscle acetone powder isolated from rabbit leg muscle was kindly provided by Prof. A. Muhlrad of the Hebrew University of Jerusalem.

Bovine catalase, 5-dimethylaminonaphthalene-1-sulfonyl (dansyl) chloride, phenyl methyl sulfonyl fluoride (PMSF), EDTA, KBr, agarose, AAPH 2,2*-Azobis(2-amidino propane) hydrochloride, horse heart myoglobin and human haptoglobin (1-1 and 2-2) were purchased from Sigma Chemical Co., St. Louis, MO, USA. Hydrogen peroxide (Merck, Darmstadt, Germany); DE-52 cellulose (Whatman International, Maidstone, England); PD-10 desalting columns (Amersham Pharmacia Biotech, Buckinghamshire, England); chemicals for SDS-PAGE (Bio-Rad Laboratories, Richmond, CA.); gases: carbon monoxide, at least 99.5%, was supplied by Gordon Gas and Chemicals Ltd., Israel, nitrogen, at least 99.9%, was supplied by the Israel Oxygen Center.

For spectrophotometric measurements a UV/vis 920 GBC, Dandenong, Australia, was used. Fluorescence measurements were carried out using Jasko FP-6200 spectrofluorimeter which allows four simultaneous kinetic measurements.

### Protein preparation

Myosin was prepared from frozen muscle acetone powder according to the available procedure literature [26]. Its concentration was determined using ε = 2.82×10^−6^ M^−1^ at 280 nm [27].

Hemoglobin was isolated from red blood cell lysates by ion-exchange chromatography using CM-52 cellulose [2] followed by desalting on PD-10 columns. This Hb was verified as oxyHb by UV-Visible absorption spectrum. Lack of catalase was certified by ferryl Hb formation in the presence of an equimolar amount of H_2_O_2_.

MetHb was prepared from the oxyHb [2] and before use it was purified by admixing with DE-52 cellulose for 5 min followed by centrifugation to remove globin-free hemin contaminants [28].

COHb was prepared by administration of carbon monoxide at atmospheric pressure for one minute and verified by its specific absorption peaks [2].

Myoglobin, which is supplied in the oxidized form, was prepared by applying commercial ferric Mb to a Sephadex G-25 column with a few grains of solid sodium dithionite spread on top to maintain Mb in its ferrous state. The immediate separation of the protein from dithionite prevented protein oxidation. The concentration of oxyMb was determined according to the literature [2].

Concentrations of all hemoproteins were expressed in heme equivalents throughout this study, except experiments which include Hp, where αβ globin (2 hemes) was considered a unit interacting with Hp.

Haptoglobin (Hp): concentrations of commercial Hp were determined by repeated measurements using the Lowry method, ε_278 nm_ = 53.90×10^−6^ M^−1^ for Hp1-1 and 58.65×10^−6^ M^−1^ for Hp2-2 (per αβ unit) [29].

LDL: native form (nLDL) was isolated from freshly drawn venous blood of healthy volunteers using established procedure [30]. To clear LDL from the KBr and EDTA added during the preparation, it was eluted through two PD-10 columns against argon-saturated phosphate buffered saline (PBS), pH 7.4. Protein concentration of isolated LDL was determined by the method of Lowry et al. [31]. nLDL was kept under argon at 0°C (an ice/water mixture) and was used up within 7 days. Vitamin E depleted LDL (dLDL) was prepared from nLDL according to the literature [32]. dLDL was kept under argon at 0°C to be used up within 24 hours.

### Analytical procedures

Heme transfer from hemoprotein to LDL was measured using FRET (fluorescence resonance energy transfer), a method which exploits fluorescence quenching due to energy transfer from protein to dansylated LDL [33]. The reaction mixtures contain dansylated LDL, H_2_O_2_ and alternately Hb and Mb. The anaerobic atmosphere was either nitrogen (control) or CO. Heme transfer kinetics was followed by observing fluorescence at 400 nm, as shown in an earlier study [6].

LDL oxidation was monitored by lipid and/or protein parameters.

LDL fractionation by charge was carried out using fast anion exchange liquid chromatography (Mono QR HR 5/5 type) according to previous literature [34].

Lipid oxidation in LDL was determined as TBARs (ThioBarbituric Acid Reactive substances) according to the literature [35], [36]. To minimize the effects of light scattering, a longer wavelength of 532 nm was measured. Additionally lipid oxidation was monitored by the formation conjugated dienes (CD) at 268 nm rather than 234 nm to minimize the effects of light scattering [6], [37].

Fluorimetric bityrosines screening: this technique allows continuous monitoring of bityrosines formation resulting from the quenching of tyrosine radicals to form bityrosines [38]. Fluorescence excitation is at 327 nm and emission at 400 nm.

SDS-PAGE analysis of cross-linked protein: oxidation products of ApoB were followed by SDS-PAGE with β-mercaptoethanol using 6–12% acrylamide bilayer slabs. Gels were stained with Coomassie Brilliant Blue R-250 [6].

### Reaction mixtures

To maintain *in vivo* conditions throughout the study, a minimal concentration of RH that might appear in plasma, 3–5 µM, was used. An equimolar concentration of hydrogen peroxide was added. All solutions were devoid of free oxygen. All reactions were carried out at 37°C in PBS, pH 7.4, unless otherwise mentioned.

## Results

Scientific literature is filled with experimental data related to Hb damage and protection. Yet most *in vitro* studies use high concentrations of peroxide, a low pH, room temperature and aerobic conditions which don't exist *in vivo*. To relate *in vitro* to *in vivo* conditions, the current study used a low peroxide concentration (µM range) as well as a lack of free oxygen (hypoxia) [25].

### Hp attenuates heme loss while allowing oxidation of ferrous Hb

The first experiment compares damage to the heme core by peroxide in the absence and presence of Hp (Type 1-1). Three reaction mixtures were examined: 1) oxyHb in buffer alone; 2) as the first plus equimolar quantities of hydrogen peroxide; 3) as the second plus Hp equimolar to oxyHb. Reaction components were added in the following order: Hb, H_2_O_2_ and Hp. All three mixtures were incubated at 37°C for 30 minutes and absorption spectra in the Soret region were recorded.

**Figure 1:**
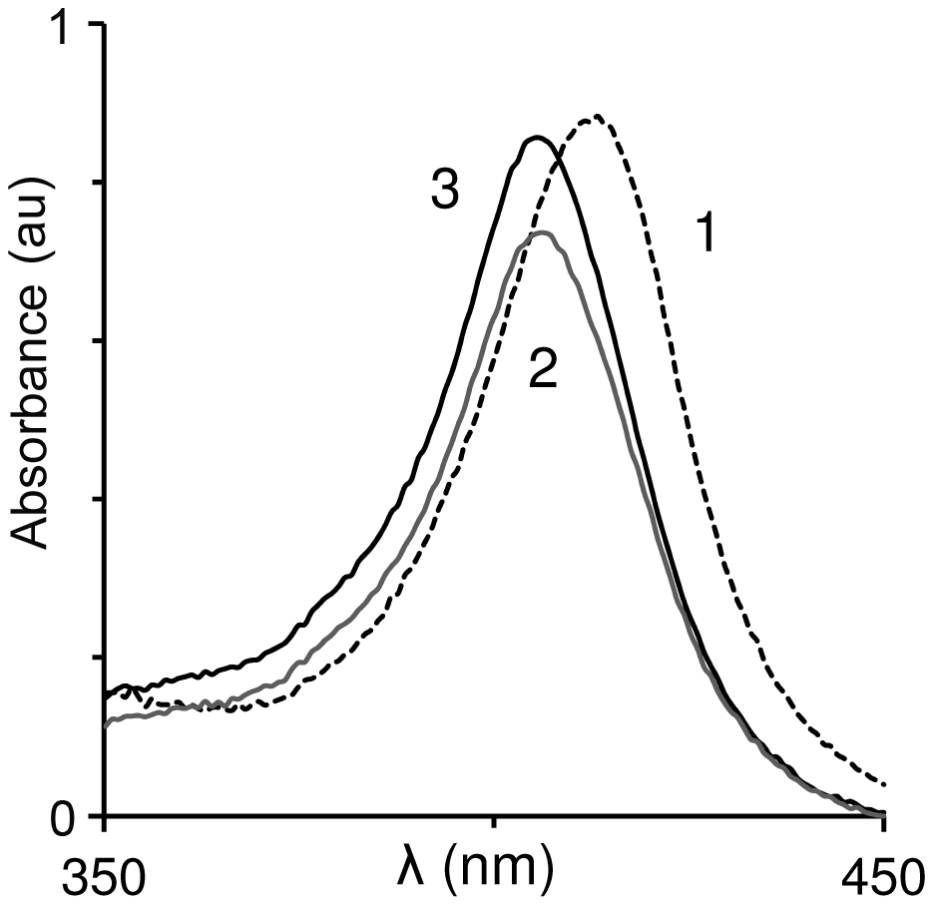
Hp attenuates heme loss while allowing oxidation of ferrous Hb. Spectrum of OxyHb (5 µM) was recorded. H_2_O_2_ (to yield 6 µM) was added and the mixture was incubated in 37°C and the spectrum recorded again. Experiment was repeated in presence of Hp1-1 (6 µM) 1: time zero (with or without Hp); 2: following 30 min in absence of Hp; 3: following 30 min in presence of Hp. Representative spectra of 3 independent experiments are shown.

As seen from the spectra (Fig. 1), H_2_O_2_ oxidized oxyHb to ferric-Hb, as indicated by a shift in the Soret peak from 414 nm to 405 nm [2]. Hp thus did not prevent ferrous to ferric iron oxidation. These findings are in correlation with previous studies carried out in air and high peroxide conditions [39]. In the two cases where the heme iron was oxidized (spectra 2 and 3), since the extinction coefficient of ferric-Hb is 1.43 times higher than that of oxyHb [2], if all the oxyHb were to undergo oxidation to ferric state, we would expect the peak to be higher. However, as seen from the spectra, some of the expected absorbance was lost, indicating that a portion of heme disintegrated. Nevertheless, the Soret peak formed in the presence of Hp (spectrum 3) is higher than the one formed in its absence (spectrum 2). This implies that the heme moiety was partially shielded from oxidative disintegration by the bound Hp, in agreement with previous data [12], [33].

### Hp inhibits Hb-induced LDL oxidation

**Figure 2:**
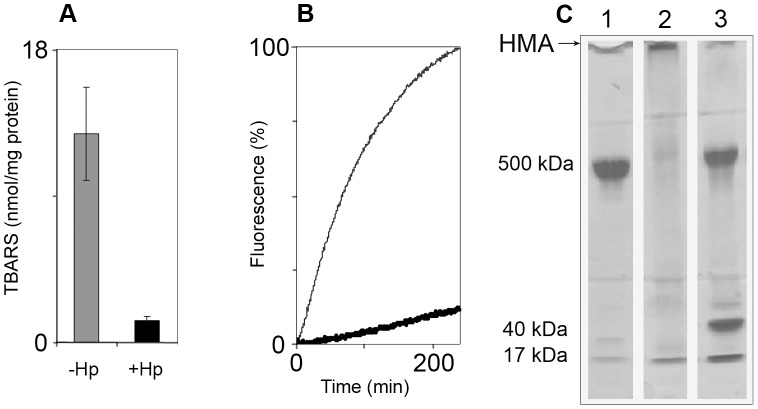
Hp inhibits Hb-induced LDL oxidation. OxyHb and H_2_O_2_ applied to freshly prepared LDL in presence and absence of Hp1-1. Different parameters known to reflect LDL oxidation were followed. A: Lipid oxidation products measured as TBARS (n = 3). Grey – LDL+oxyHb+H_2_O_2_; Black – LDL+oxyHb+H_2_O_2_+Hp. Concentrations: LDL – 600 µg protein/ml; oxyHb – 3 µM; H_2_O_2_ – 3 µM; Hp – 5 µM. Time of incubation – 3 hours. B: Kinetics of bi-tyrosine formation. Fine line – LDL+oxyHb+H_2_O_2_; Heavy line – LDL+oxyHb+H_2_O_2_+Hp. (Ex/Em = 327/400 nm). Concentrations: LDL – 100 µg protein/ml; oxyHb – 3 µM; H_2_O_2_ – 3 µM. A representative result of 3 independent experiments is shown. C: SDS-PAGE: Formation of high molecular-weight aggregates – HMA. 1: oxyHb+LDL; 2: oxyHb+LDL+H_2_O_2_; 3: oxyHb+Hp+LDL+H_2_O_2_. Concentrations: LDL – 600 µg protein/ml; oxyHb – 3 µM; H_2_O_2_ – 3 µM; Hp – 5 µM. A representative result of 3 independent experiments is shown.

As seen in the previous experiment, a portion of heme disintegrated in solution. This portion may oxidatively attack neighboring components. To check if this is the case, we measured the oxidation of both protein and lipids in LDL under the same conditions as before. LDL was chosen as an *in vivo* substrate in circulation that is sensitive to oxidation by cell-free Hb. A mixture of Hb and H_2_O_2_ was incubated with LDL for three hours at 37°C, under nitrogen. Fig. 2A shows lipid oxidation quantified by the formation of TBARS. Comparing lanes 1 and 2 in Fig. 2A indicates that the association of Hb with Hp1-1 inhibited the formation of lipid peroxides. Protein oxidation was observed kinetically by examining the fluorescence of bityrosines formed in LDL. An example for differences in oxidation when ferrous Hb is employed can be seen in a previous study from our laboratory [40]. Fig. 2B shows that Hp almost fully inhibits the reaction. Bityrosines that form may be either intramolecular or intermolecular. As only one protein molecule exists per LDL particle, intermolecular cross-linking results in aggregation.

An SDS-PAGE was run in order to analyze the aggregation state of the protein. Lanes 1 and 2 in Figure 2C indicate that within 3.5 hours, little protein remained as the original 500 kDa monomer (apoB). Most of the protein had become cross-linked in a covalent intermolecular form (HMA – High Molecular-weight Aggregates). In contrast, in the reaction mixtures containing Hp, most of the protein remained as an apoB monomer, indicating that the presence of haptoglobin was able to prevent the formation of covalent aggregates of LDL.

### A Hp with reduced activity prevents oxidation of dLDL

There are certain *in vivo* conditions in which LDL is more vulnerable to oxidation. One condition is a lack of vitamin E within the particle, a vitamin E-depleted form (dLDL), rendering it more sensitive to oxidation. Another condition is a type of haptoglobin which has a weaker protective effect against hemin release, Hp 2-2 [41]. To evaluate the degree of protection from cell-free Hb provided by Hp to the vasculature, we tested whether Hp 2-2 could protect dLDL. Ferric-Hb was incubated with dLDL, with and without Hp 2-2. As a double control, dLDL and Hp were each incubated alone. As dLDL is highly negatively charged, an agarose column was used to separate the components of the reaction mixtures according to charge. The column was developed using a salt gradient of 0–1 M NaCl, buffered with tris-HCl at pH 7.4, and protein fractions were eluted.

**Figure 3:**
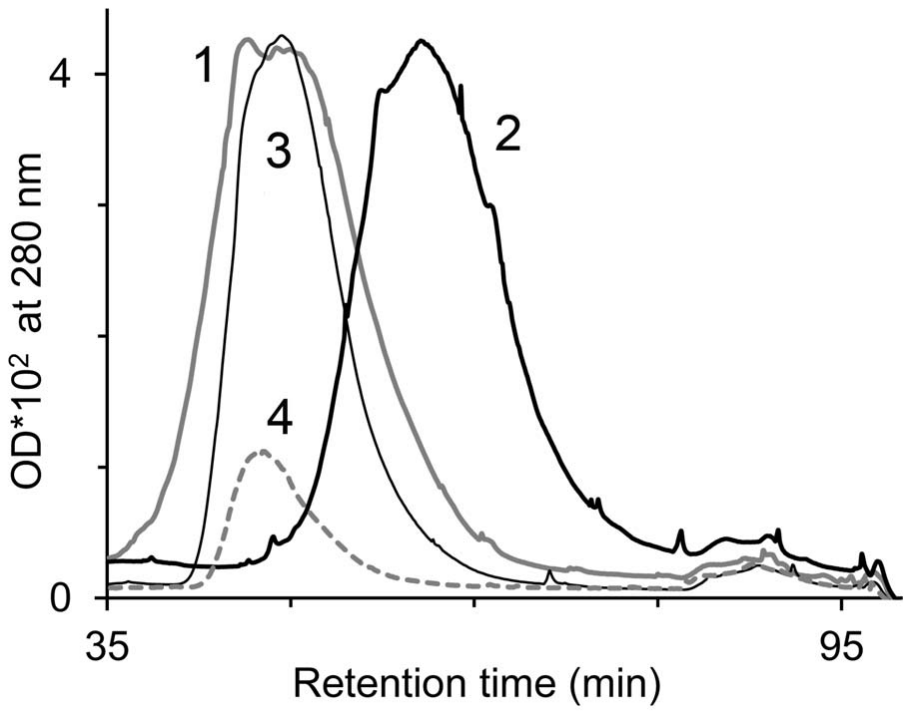
Haptoglobin 2-2 inhibits dLDL oxidation. A solution containing dLDL (1.2 mg protein/ml) and ferric-Hb (12 µM) was incubated in presence or absence of Hp 2-2 (13 µM). LDL fractionation by charge following 3.5 hours of incubation was performed by fast anion exchange liquid chromatography. 1: dLDL alone (control); 2: dLDL+ferric Hb; 3: dLDL+ferric Hb+Hp; 4: Hp alone (control). A representative result of 4 independent experiments is shown.

As seen from Fig. 3, the elution peaks of Hp alone (peak 4) and LDL alone (peak 1) appear at ∼50 minutes, while the elution peak of oxidized LDL (peak 2) was at ∼60 minutes. This may be due to an increase in negative charge on LDL, caused by its oxidation. In contrast, the presence of Hp in the reaction mixture caused the elution peak to remain at LDL's original position (peak 3).

### CO attenuates Hb-induced LDL oxidation

In a previous study, we showed that the mutual presence of CO and peroxide arrests ferric-Hb in the carboxy, redox-inactive, ferrous form [24]. In the first part of the current study, we stated that oxidation of LDL takes place even under an anaerobic atmosphere. The following experiment determined whether ferric-Hb can oxidize LDL in a CO atmosphere.

**Figure 4:**
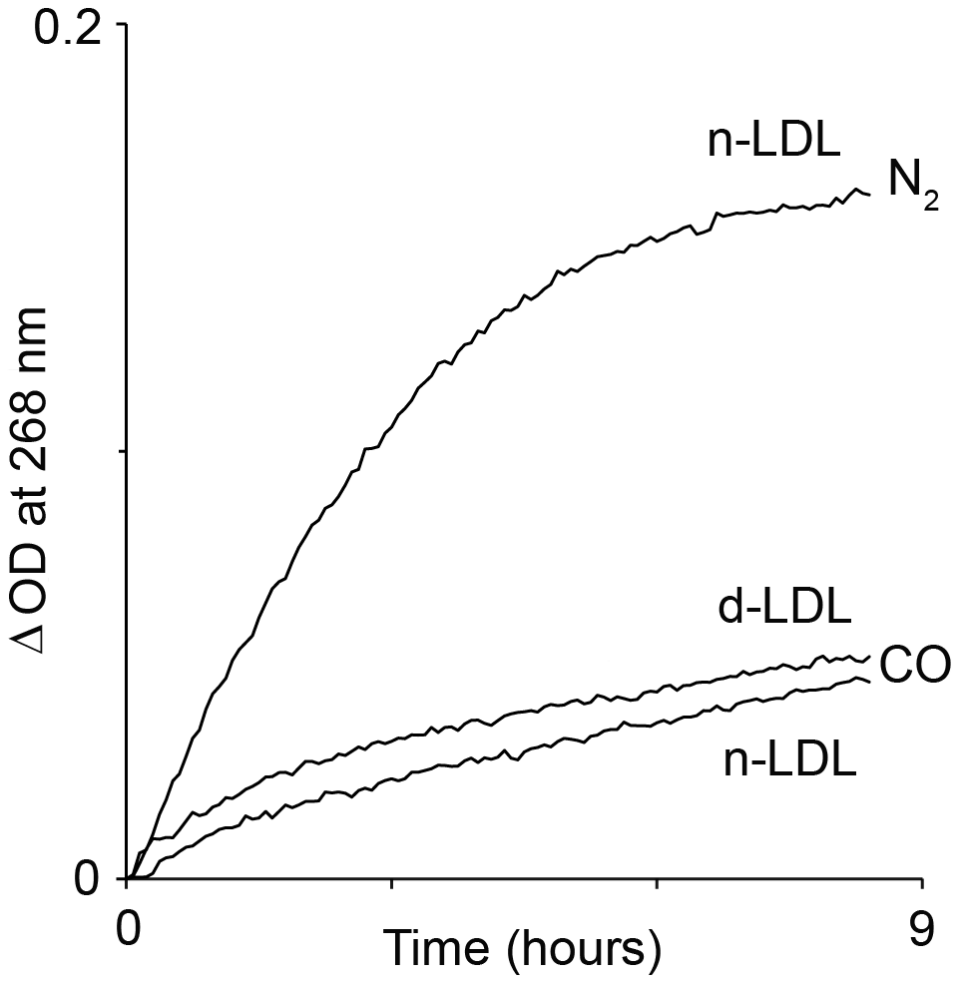
CO attenuates Hb-induced LDL oxidation. LDL oxidation kinetics was traced in reaction mixtures containing ferric-Hb (3 µM), nLDL or dLDL (100 µg protein/ml) and H_2_O_2_ (3 µM) at anaerobic conditions of N_2_ or CO. Protein oxidation was followed by formation of conjugated dienes. Reactions rate is shown as ΔOD since the proteins (LDL and Hb) contribute to light absorption in UV region (ΔOD = OD(t_x_)−OD(t_0_) ). A representative result of 3 independent experiments is shown.

LDL oxidation kinetics was traced in reaction mixtures containing ferric-Hb, LDL, and hydrogen peroxide. Air was replaced with either N_2_ or CO. Protein oxidation was observed by the formation of conjugated dienes at 268 nm. Fig. 4 shows that the rates of Hb-induced oxidation in the presence of CO were reduced dramatically. Oxidation under CO proceeded more slowly. Suppression of oxidation by CO also occurred when the freshly prepared LDL was replaced by dLDL, although to a lesser extent.

### Effect of CO on heme transfer from ferric-RH to LDL

**Figure 5:**
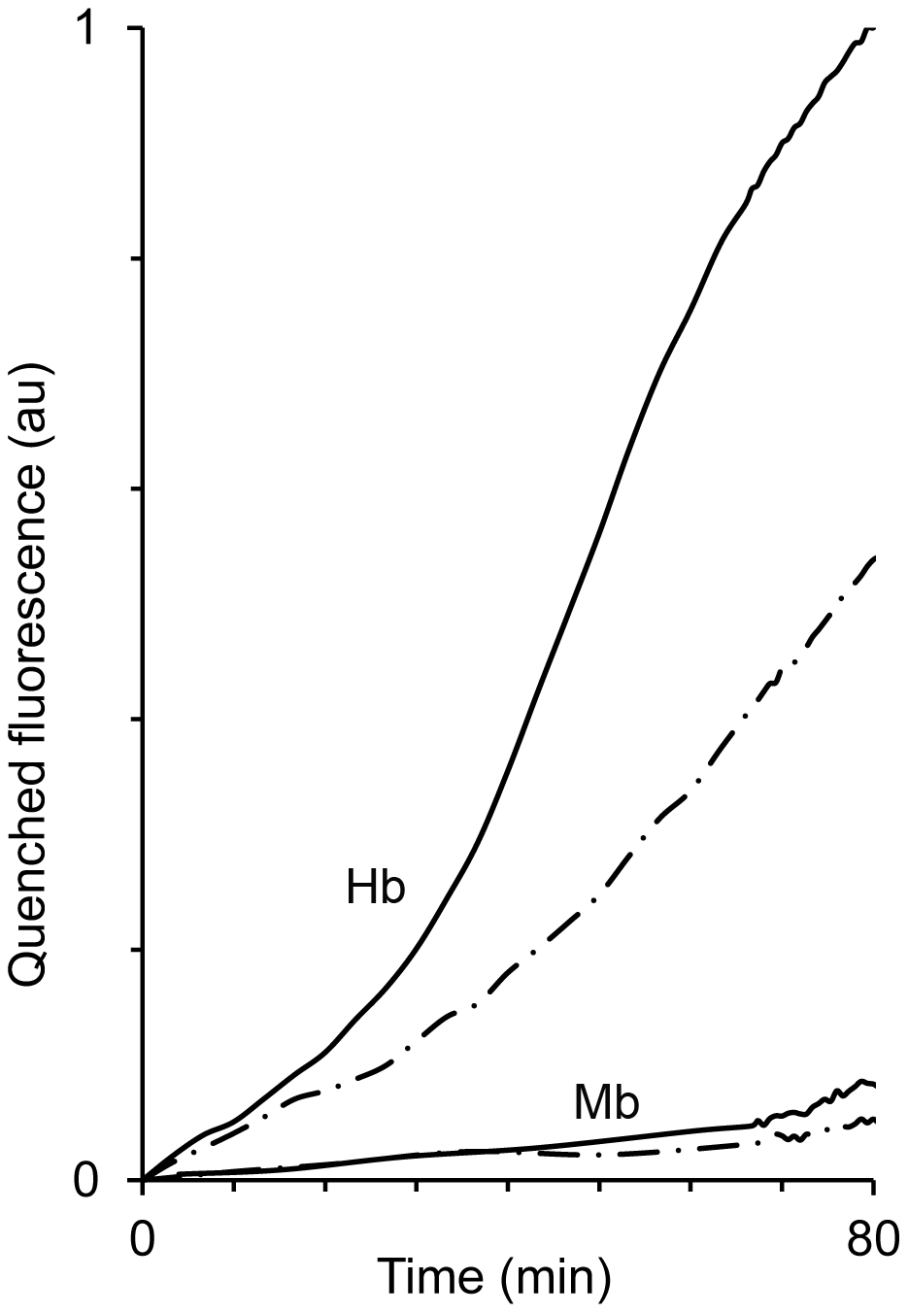
Effect of CO on heme transfer from ferric-RH to LDL. The rate of heme transfer to LDL was traced in reaction mixtures containing either Hb or Mb (3 µM), dansylated LDL (100 µg protein/ml) and H_2_O_2_ (3 µM) at anaerobic conditions provided by N_2_ (solid line) or CO (dot-dash line). The two upper lines – Hb and the two lower lines – Mb. (Ex/Em = 327/400 nm). A representative result of 4 independent experiments is shown.

As shown above, CO can diminish the oxidation of LDL by Hb. We wished to find out whether Hb-induced LDL oxidation is the direct outcome of intact hemoglobin or globin-free heme. To evaluate the heme transfer from globin to LDL, ApoB was dansylated, according to a previous study from our lab [6]. Heme that binds to LDL results in quenching of fluorescence of the dansyl probe. The results in Fig. 5 show that carbon monoxide appreciably diminishes the rate of heme transfer to LDL. In comparison, Mb exhibited only negligible quenching, with a further reduction of transfer under CO. This finding corresponds with results carried out previously [6].

### CO locks HRP iron in a ferrous state

**Figure 6:**
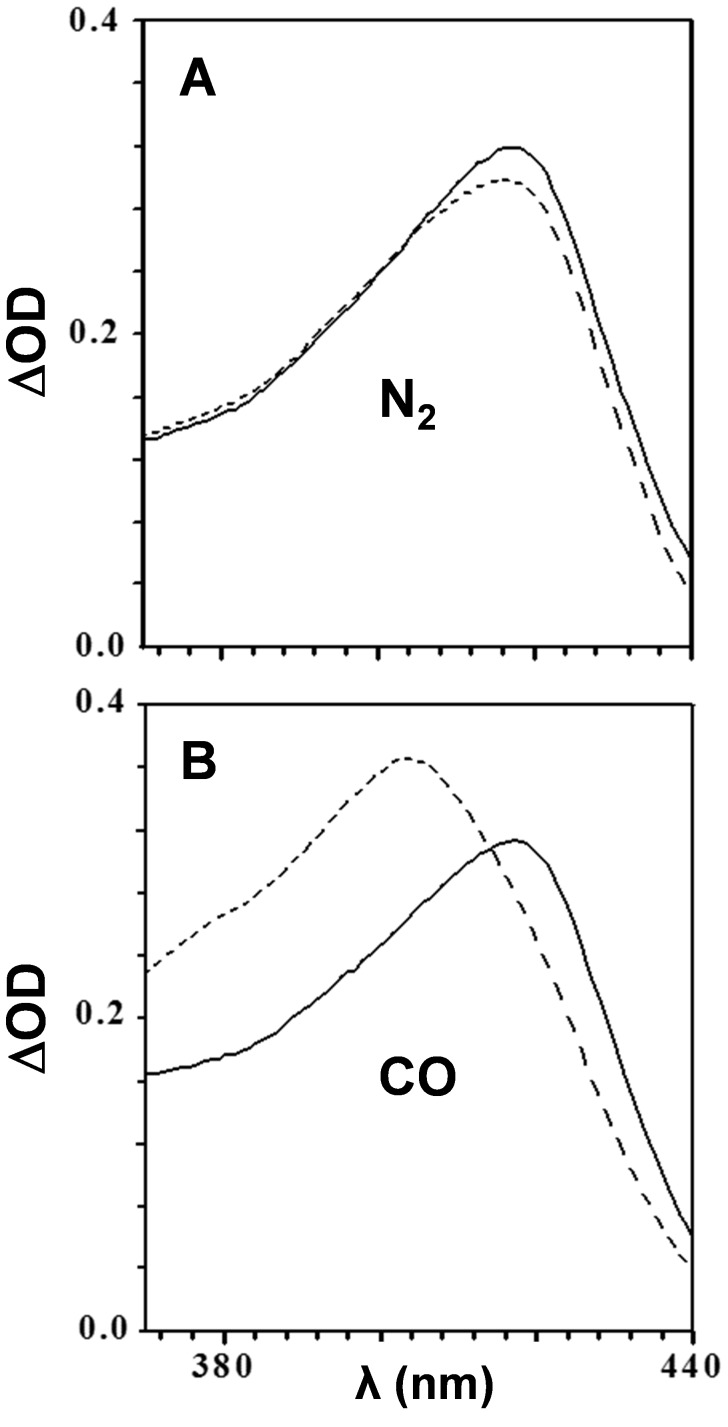
Soret absorption spectra of peroxide-activated HRP in anaerobic atmospheres. HRP (3 µM) was mixed with H_2_O_2_ (100 µM) and LDL (100 µg protein/ml) at anaerobic conditions provided by either N_2_ (A) or CO (B). Soret region absorption spectra were recorded at time zero (solid line) and following 60 min (dashed line) of incubation at 37°C. A representative result of 3 independent experiments is shown.

Previously, it was reported that HRP was completely unable to peroxidize LDL in the presence of CO [42], but no mechanism was suggested. We showed in the current study that Mb does not release heme easily, operating as an intact enzyme (Fig. 5). In Fig. 6, we show absorption spectra of HRP in the Soret region of reaction mixtures containing CO and peroxide at time zero and after one hour's incubation. Fig. 6A depicts practically no change in the Soret peak of the heme following incubation in nitrogen. In contrast, Fig. 6B demonstrates that under CO, ferric heme was reduced to carboxy-ferrous heme, locking the enzyme in a redox inactive state. This mechanism provides an explanation for the previous observation that LDL oxidation is arrested in the presence of CO [42].

### Mb peroxidase-like redox activity is specifically restrained by CO

**Figure 7:**
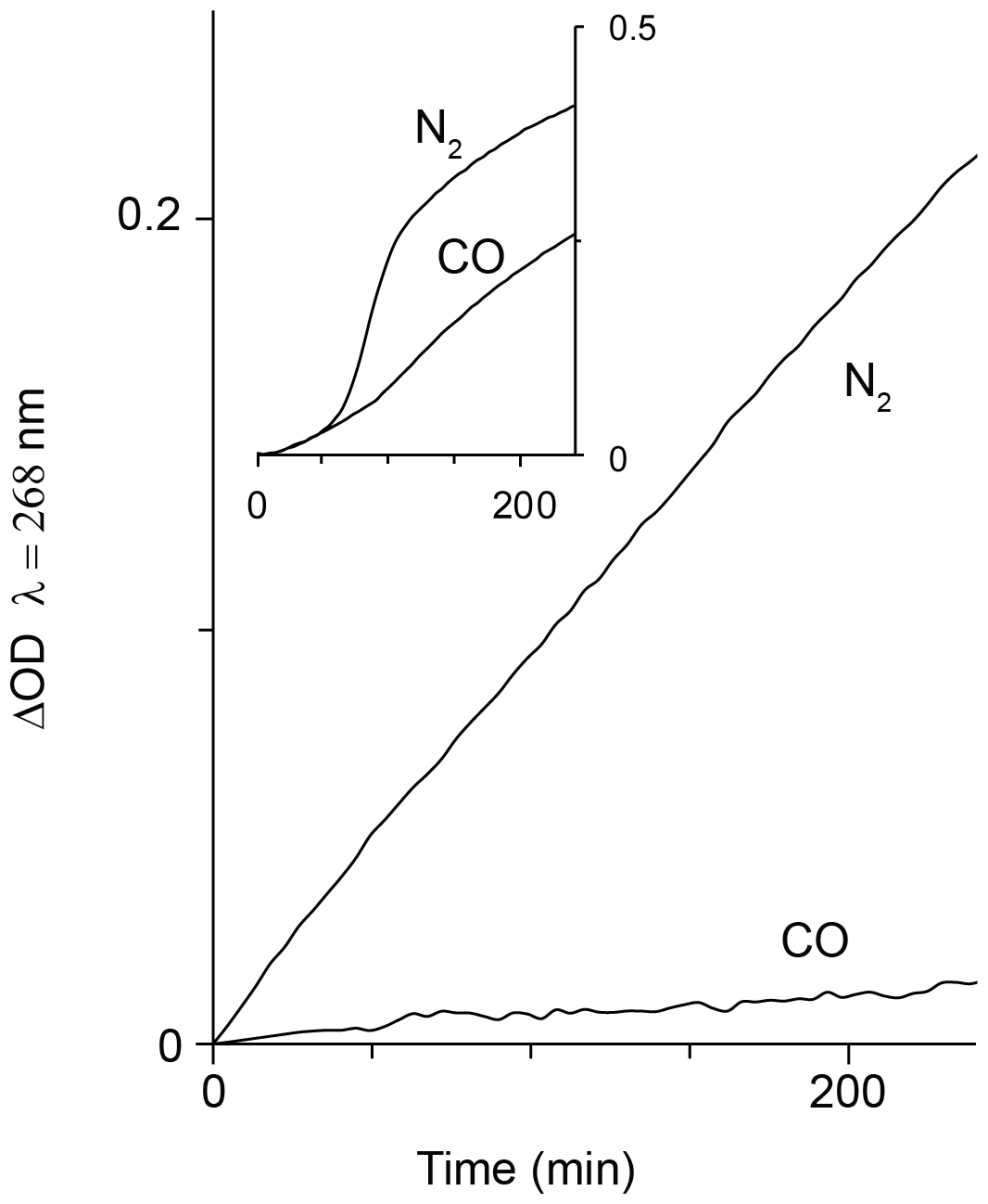
RH-induced LDL peroxidation under anaerobic atmospheres. The rate of LDL oxidation was traced in reaction mixtures containing either Hb or Mb (3 µM), LDL (100 µg protein/ml) and H_2_O_2_ (3 µM) at anaerobic conditions provided by N_2_ or CO. (ΔOD = OD(t_x_)−OD(t_0_) ) Main figure – Mb; insert – Hb. A representative result of 3 independent experiments is shown.

As reported previously, of the two RH proteins Mb is closer in its enzymatic function to HRP than Hb [6]. Therefore, it was of interest to compare the effect of CO on Mb (main figure, Fig. 7) and Hb (insert) oxidative activity. The kinetics of LDL oxidation by Hb or Mb was traced under a nitrogen or CO atmosphere. It appears that under nitrogen, Mb induced the oxidation of LDL at a uniphasic rate, as typified by enzyme kinetics. On the other hand, the insert indicates that Hb has a completely different multiphasic kinetic pattern, resulting from the activity of heme that was released from the globin. Finally, under CO, the oxidative activity of Mb was almost completely arrested. This stands in sharp contrast to the effect of CO on Hb, where oxidation still took place, albeit at a slower rate.

As stated in the introduction, an important difference between Hb and Mb is the physiological environment in which each RH resides and operates. Mb resides in the cell, dominated by other proteins. Therefore, in the next experiment, we traced the effect of Mb on myosin under oxidative conditions.

### CO blocks Mb-induced myosin oxidation

**Figure 8:**
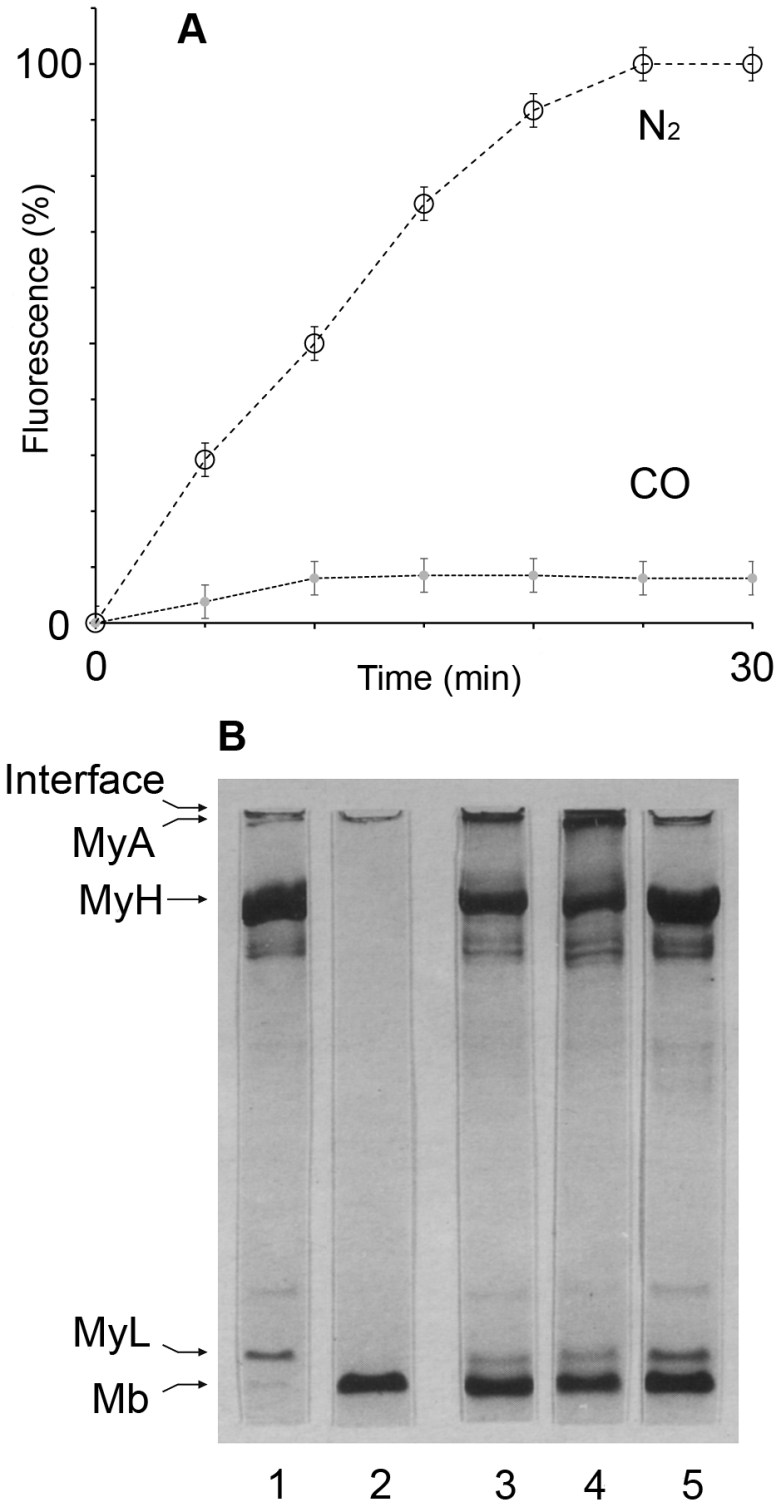
CO blocks Mb-induced myosin oxidation. A: Myosin oxidation is followed by fluorescence intensity of bityrosines formed. Reaction mixtures contained My (0.2 µM), oxyMb (0.5 µM) and H_2_O_2_ (200 µM). (25°C, Ex/Em = 327/410 nm, n = 5.); B: Myosin (2.5 µM) oxidation by Mb is followed by formation of covalent high molecular weight aggregates as demonstrated by SDS-PAGE. 1: My alone and also in presence of H_2_O_2_ (control). 2: Mb alone and also in presence of H_2_O_2_ (control). 3–5: oxyMb (30 µM)+H_2_O_2_ (200 µM); pH 7.3; 40 min incubation in air (3); under N_2_ (4); under CO (5).

OxyMb was mixed with excess hydrogen peroxide in order to peroxidize myosin. The formation of bityrosines was traced by its fluorescence emission as detailed in a previous study [38]. As seen from Fig. 8A, under nitrogen the reaction reached completion after 25 minutes. In contrast, under CO, a negligible amount of bityrosines formed, and the reaction reached completion after ∼10 minutes. To analyze which components of the mixture took part in the formation of bityrosines, reaction mixtures were incubated for 40 minutes to reach completion and analyzed with SDS-PAGE. As seen in Fig. 8B, the reaction resulted in the reduction of the myosin bands in favor of high molecular weight bands at the gel interface (lane 3). These bands, as shown in previous literature [43], represent intermolecular covalent aggregates. Evidently, myosin monomers (heavy and light chains) faded while aggregates were seen to appear more strongly. Replacing air with nitrogen resulted in practically the same bands, as shown in lane 4. However, replacing nitrogen with CO resulted in a complete inhibition of aggregation, as seen from the gel: bold myosin monomer bands in lane 5 are favored over weak bands of aggregates.

## Discussion

The study shows that in the absence of free oxygen and at low peroxide levels, ferrous-Hb undergoes oxidation to ferric-Hb. Under such conditions, hemin disintegrates rapidly, judging by the partial loss in absorbance of the ferric Soret band. In corroboration with a previous study [12], while the association of Hb with Hp did not inhibit the oxidation of ferrous to ferric iron, hemin was protected from disintegration. The current study further demonstrates that when a solution of cell-free Hb contains circulatory components of plasma, like hydrophobic LDL, hemin readily transfers from globin to these components. As a result, both lipids and the apoB protein of the LDL particle undergo oxidation (Fig. 2). Hp acts by trapping the heme in the Hp-Hb complex, such that it can no longer oxidize LDL. The Inhibition of heme-induced oxidation even holds true in the case of the less effective form, Hp 2-2, and the vulnerable form of LDL, dLDL (Fig. 3). These results clearly show that the efficiency of Hp in inhibition of Hb oxidative activity stems from preventing sensitive targets from associating with loosely-bound hemin [44]. The specific protection afforded to Hb by Hp fits in well with our knowledge of the dissociation of Hb into αβ dimers at low concentrations occurring *in vivo*. Hp prevents the escape of the loosely-bound hemin since it masks the surface of the αβ dimer when it binds, covering a large part of the Hb interface [45]. The current study indicates that CO provides some protection against oxidation of LDL by Hb, but this protection is much less efficient than that of Hp.

**Figure 9:**
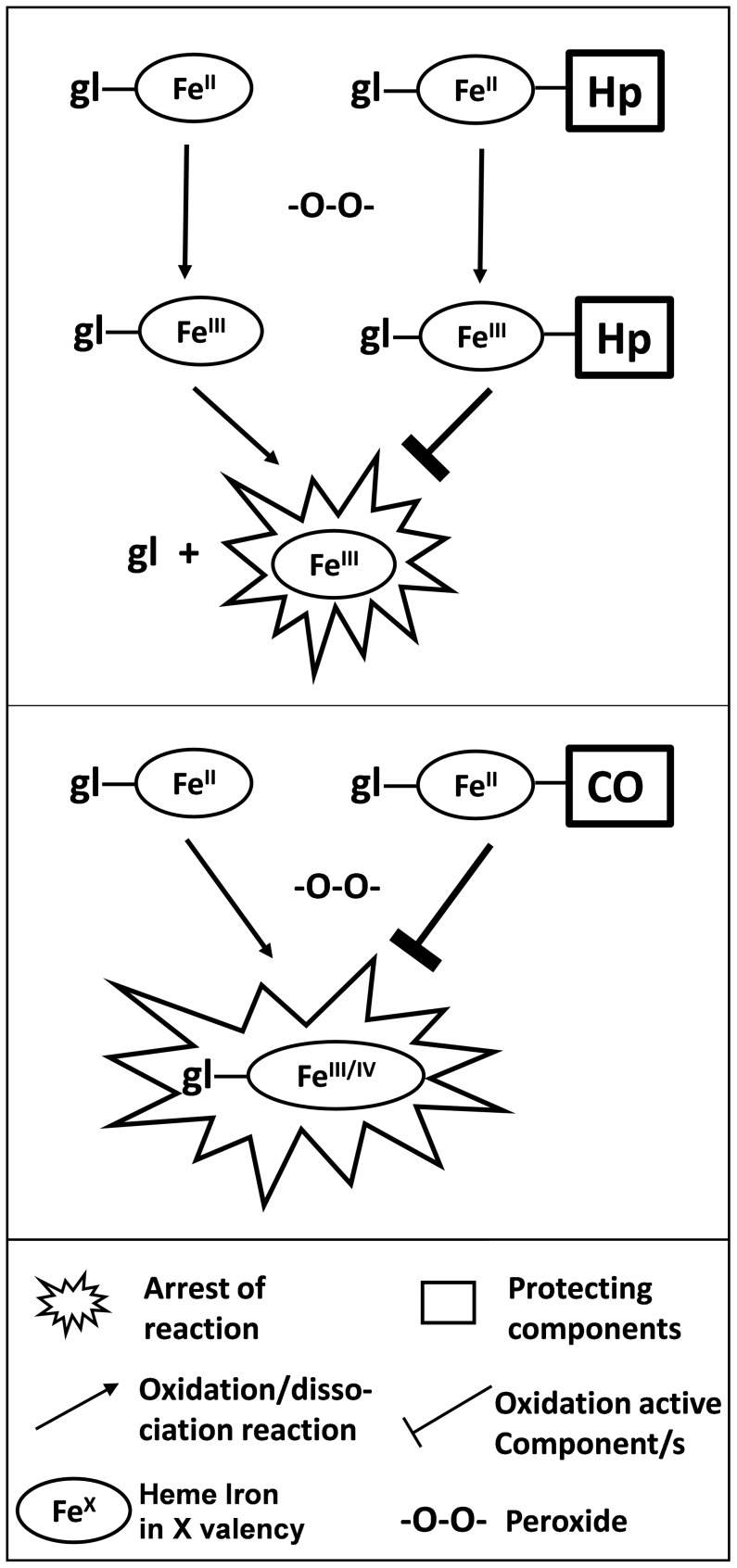
Differences in Hb and Mb induced oxidation yield distinct protection mechanisms. In presence of peroxide, ferrous RH are oxidized to their ferric (Fe^III^) and/or ferryl (Fe^IV^) forms. Upper: Hp binds Hb (ferrous and/or ferric) thereby preventing its release. Lower: Mb heme is retained attached to globin following oxidation in a peroxidase-like form. However, binding of CO to ferrous Mb prevents its oxidation to a ‘peroxidase-like form”.

The prominent difference between the two mechanisms by which Hb and Mb act, relates to the fact that heme transfer from Mb is negligible in comparison to Hb (Fig. 5). Differences in mechanisms of the two proteins are especially prominent when observing the kinetics of LDL oxidation and its arrest by CO (Fig. 7, main). LDL is oxidized by ferric-Mb at a constant rate, appropriate for enzymatic function. This differs completely from the multistage rate of Hb-induced LDL oxidation (Fig. 7 insert). Despite a common physiological function (oxygen binding to a divalent heme iron), Hb and Mb differ in the mechanism by which they evoke oxidation. Hb oxidative activity results fundamentally from a weakening of the trivalent heme-globin bond [44]. As discussed earlier, ferric-Hb redox activity is manifested by the presence of components that bind hemin strongly, such as LDL [6], [33]. Thus, only a high–affinity globin–binding protein, such as Hp, can efficiently trap hemin. On the other hand, as suggested in the past and indicated in the current study, Mb's oxidative power stems from a protein-bound ferric heme whose activity is peroxidase-like: namely, fully dependent on heme iron redox capability. The differences in mechanism of oxidation induced by the two RH proteins are demonstrated schematically in Fig. 9.

As mentioned in the introduction, besides differences in structure/function, the physiological compartment in which RH are found is completely disparate. Hb is short-lived and becomes harmful when expelled from a ruptured cell to the vasculature. Mb, on the other hand, resides in a long-lived, nucleated myocyte, filled with functional proteins and thus may cause oxidative damage both inside and outside the myocyte.

Single-chained extra-cellular Mb deposits in the kidney, causing major damage there. In addition to the large amount of Mb, some Hb also finds its way into the kidney following rhabdomyolysis. It was shown in a rat rhabdomyolysis model that the presence of residual amounts of Hb aided in countering an overdose of Mb, causing oxidative damage [22]. These conditions induce HO-1. Recent literature in combination with the current study suggest the following explanation: ferric-Hb readily liberates globin-free hemin, an inducer of HO-1. The product of the enzymatic reaction, CO, spreads to the immediate vicinity, binding strongly to ferrous Mb, rendering it inactive. This is summarized below:
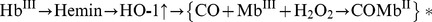
{}* - combined reducing effect of CO and peroxide exhibited in a previous study [24].

The minute amount of hemin dispelled from Mb in the cell induces production of HO-1 which produces more CO. In the myocyte, Mb resides in close proximity with contractile proteins that are prone to oxidation. Therefore, their immediate protection from injury induced by ferric-Mb is essential.

We and others demonstrated in previous studies that under oxidative stress, Mb triggers the oxidation of myosin followed by its cross-linking. These alterations to protein structure are associated with a variety of pathologies. Moreover, aggregated myosin loses its ATPase activity [43], [46].

Central to oxidative injury to striated muscle is ischemia/reperfusion (I/R). It has been shown already a decade ago that CO added prevents I/R injury [47]. Reperfusion injury is the damage caused to tissue, such as muscle, after the blood supply is cut off and later returns. It has been established that overexpression of HO-1 protects the heart from I/R injury [48]. A recent study described protection by CO-RH but the mechanisms involved are still unclear [49]. Based on the current study, we suggest that induction of HO-1 by hemin, yields the product CO. The gas will diffuses fast out to occupy a vacant ferrous iron site on Mb, preventing adverse oxidation.

Other cases of induced HO-1 are related to muscle disuse atrophy. Up-regulation of HO-1 was revealed unexpectedly by the authors during an experimental model inducing disuse atrophy in rats [50]. Based on the findings of the current study, it can be anticipated that as a result of heme dismantling by HO-1, the antioxidant product, CO, arrests the oxidative activity of Mb.

Ordinarily, following strenuous activity, the muscle cell loses a considerable amount of its Mb-bound oxygen, leaving part of it in a deoxy state. Deoxy-Mb is less stable and more active in inducing oxidation [51], [52] and thus harmful. As stated above, under oxidative conditions myoglobin releases a fraction of its hemin, inducing HO-1, which in turn releases CO during heme catabolism. A shortfall in free oxygen leads to the immediate saturation of deoxy-Mb with CO. Carboxy-Mb is a most desired complex for the provisional preservation of Mb as ferrous-Mb. In support of the above-suggested scenario, a previous study of skeletal muscle types pointed out that induction of HO-1 follows a fiber-type specific pattern such that upon addition of free hemin, induction is more pronounced in red muscle with a high Mb content [53].

In this context, it has been shown that inhaled carbon monoxide protects muscles from structural injury and energy depletion following I/R, as well as modulates inflammation in tissue after therapy, without affecting the production of HO-1, providing a form of metabolic rescue [54].

Taking together the current and our previous study [6], it appears that hemin binds more strongly to Mb than to Hb. The transfer of heme to LDL is consequently different in the two molecules. In Hb, hemin is trapped by ApoB protein and thereafter disintegrates in conjunction with LDL oxidation. Unlike Hb, where the transferred heme triggers oxidation, Mb acts as an intact hemoprotein peroxidase, resembling HRP. Mb's heme iron redox is based on Fe^III/IV^ activity. Thus, in order to block redox activity of Hb, a chaperone protein, Hp, is required, while in order to block injurious Mb redox activity, a small, yet efficient electron trap will suffice. The specific and high affinity of CO for ferrous iron beckons its call. As shown in a previous study [24], CO defused the peroxide and arrested the heme iron in a ferrous state.
