# Correction: Can Gas Replace Protein Function? CO Abrogates the Oxidative Toxicity of Myoglobin

**DOI:** 10.1371/journal.pone.0111565

**Published:** 2014-10-15

**Authors:** The PLOS ONE Staff

Figure 9, “Differences in Hb and Mb induced oxidation yield distinct protection mechanisms,” is incorrect. Please see the corrected Figure 9 here.

**Figure 9:**
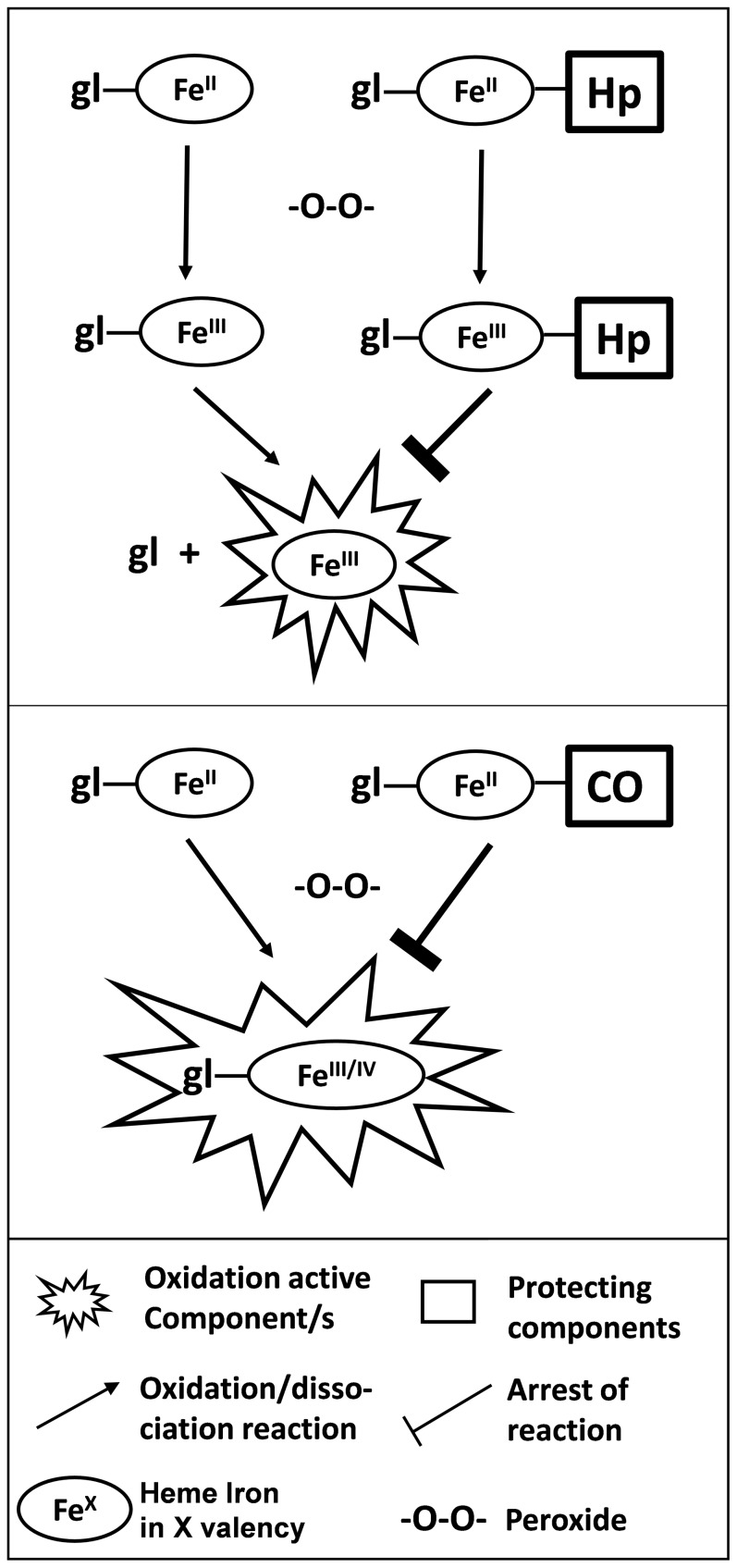
Differences in Hb and Mb induced oxidation yield distinct protection mechanisms. In presence of peroxide, ferrous RH are oxidized to their ferric (Fe^III^) and/or ferryl (Fe^IV^) forms. Upper: Hp binds Hb (ferrous and/or ferric) thereby preventing its release. Lower: Mb heme is retained attached to globin following oxidation in a peroxidase-like form. However, binding of CO to ferrous Mb prevents its oxidation to a ‘peroxidase-like form”.
